# Comparative Transcriptomes Profiling of Photoperiod-sensitive Male Sterile Rice Nongken 58S During the Male Sterility Transition between Short-day and Long-day

**DOI:** 10.1186/1471-2164-12-462

**Published:** 2011-09-25

**Authors:** Wei Wang, Zhenwei Liu, Zhibin Guo, Gaoyuan Song, Qin Cheng, Daiming Jiang, Yingguo Zhu, Daichang Yang

**Affiliations:** 1 State Key Laboratory of Hybrid Rice, College of Life Sciences, Wuhan University, North Road of Luoshi, Wuhan, Hubei Province, China

**Keywords:** transcriptome profiling, PGMS, male sterility transition, circadian rhythm, flowering

## Abstract

**Background:**

Photoperiod-sensitive genic male sterile (PGMS) rice, Nongken 58S, was discovered in 1973. It has been widely used for the production of hybrid rice, and great achievements have been made in improving rice yields. However, the mechanism of the male sterility transition in PGMS rice remains to be determined.

**Results:**

To investigate the transcriptome during the male sterility transition in PGMS rice, the transcriptome of Nongken 58S under short-day (SD) and long-day (LD) at the glume primordium differentiation and pistil/stamen primordium forming stages was compared. Seventy-three and 128 differentially expressed genes (DEGs) were identified at the glume primordium differentiation and pistil/stamen primordium forming stages, respectively. Five and 22 genes were markedly up-regulated (≥ 5-fold), and two and five genes were considerably down-regulated (≥ 5-fold) under SD during the male sterility transition. Gene ontology annotation and pathway analysis revealed that four biological processes and the circadian rhythms and the flowering pathways coordinately regulated the male sterility transition. Further quantitative PCR analysis demonstrated that the circadian rhythms of *OsPRR1, OsPRR37, OsGI, Hd1, OsLHY *and *OsDof *in leaves were obviously different between Nongken 58S and Nongken 58 under LD conditions. Moreover, both *OsPRR37 *and *Hd1 *in the inflorescence displayed differences between Nongken 58S and Nongken 58 under both LD and SD conditions.

**Conclusion:**

The results presented here indicate that the transcriptome in Nongken 58S was significantly suppressed under LD conditions. Among these DEGs, the circadian rhythm and the flowering pathway were involved in the male sterility transition. Furthermore, these pathways were coordinately involved in the male sterility transition in PGMS rice.

## Background

Photoperiod-sensitive genic male sterile (PGMS) rice, Nongken 58S, was discovered as a spontaneous mutant in the japonica rice cultivar Nongken 58 (*Oryza sativa *ssp. *japonica*) grown in Hubei Province, China in 1973 [1]. It has since been used for production of hybrid rice, and during the past two decades, great achievements have been made in improving rice yields in China using two-line hybrid rice. Several important features of Nongken 58S have been characterised. Its fertility is highly regulated by day length at specific inflorescence developmental stages. Complete male sterility can be induced when the day length is greater than 14 h from the glume primordium differentiation stage to the pollen mother cell forming stage. However, male fertility returns gradually when the day length is shorter than 14 h [1]. This is called the second photoperiod phenomenon [2], referring to the short day photoperiod that rice requires for the transition from vegetative growth to reproductive growth [2]. This agronomic trait is genetically controlled by a recessive locus within the nuclear genome. However, the genetic loci vary depending on the genetic background of the recipient parents. That is, when crossed with a less photoperiod-sensitive cultivar, the genes for the trait appear as two or three loci [3-7]. The photoperiod sensitivity can also be affected by temperature when the *pms *gene is crossed into a temperature-sensitive cultivar. Previous studies have indicated that phytochromes and cryptochromes are involved in the male sterility transition [2]. Because male sterility is highly and coordinately regulated by day length and temperature, this leads to difficulty in the accurate identification of the male sterile phenotype in the segregation populations between *japonica *and *indica *under natural conditions. Although it would be of great value to breeders, this makes mapping the *pms *gene challenging [3-5,8,9]. It is important to understand the molecular mechanism of the male sterility transition and to explore whether the light or circadian rhythm signal transduction pathway is involved in this process. It may also be useful to understand how light or the circadian rhythm regulates microsporocyte development. This information could assist breeders in selecting recipient parents for molecular breeding programs.

Circadian rhythms, in general, take the form of sinusoidal waves that can be described using mathematical terms such as period, phase and amplitude [10-12]. The expression of several genes has been found to be associated with the circadian rhythm in *Arabidopsis*. These include *TIMING OF CAB EXPRESSION1 *(*TOC1*) [13], *LATE ELONGATED HYPOCOTYL *(*LHY*) [14], *CIRCADIAN AND CLOCK ASSOCIATED1 *(*CCA1*) [15] and *GIGANTEA *(*GI*). *TOC1, LHY, CCA1 *and unknown factor Y [16,17] comprise interlocked transcriptional feedback loops. These feedback loops play important roles in the plant central clock. These loops integrate environmental factors, such as light and temperature, into the central clock through the input signaling pathway and import the rhythm signal into downstream signaling pathways through output signaling pathways.

Two main pathways controlling flowering time are found in rice. One is the *EARLY HEADING DATE1 *(*Ehd1*)/*HEADING DATE3a *(*Hd3a*)/*RICE FLOWERING LOCUS T1 *(*RFT1*)-dependent pathway [18-22]. In this pathway, the upstream gene *Ehd2*/*RID1*/*OsId1*, under short-day (SD) conditions, activates *Ehd1. Ehd1 *then activates *Hd3a*/*RFT1 *to promote rice flowering. By contrast, *Ehd1 *is suppressed under long-day (LD) conditions. *Hd3a*/*RFT1 *is also suppressed, leading to the inhibition of rice flowering [21]. The other pathway is the *HEADING DATE1 *(*Hd1*)-*Hd3a*/*RFT1*-dependent pathway [18,23]. In this pathway, *Hd1 *is activated by *OsGI *under LD or SD conditions. *Hd1 *then activates *Hd3a*/*RFT1 *expression and promotes flowering under SD conditions. However, *Hd1 *suppresses *Hd3a*/*RFT1 *expression and inhibits flowering under LD conditions [18,24,25]. Previous research has indicated that circadian rhythms and day length are not only involved in the promotion of reproductive organs from the vegetative stage in higher plants [11,26,27], but are also involved in various physiological processes, including photosynthesis [28,29], starch metabolism [30-32], phytohormone response [32-35], hypocotyl elongation [36,37], and plant-pathogen interaction [38]. However, the involvement of these two signaling pathways in the male sterility transition has not been reported.

This paper reports the comparison of the transcriptomes of Nongken 58S under SD and LD conditions during the male sterility transition. The repressive expression profile under LD conditions identified 183 differentially expressed genes (DEGs). Gene ontology (GO) and pathway analysis of the DEGs revealed that the circadian rhythm and the flowering pathways were involved in the male sterility transition. In further analysis, qPCR results indicated that the circadian rhythms of *OsPRR1, OsPRR37, OsGI, Hd1, OsLHY *and *OsDof *in Nongken 58S were significantly different from those in Nongken 58. This suggests that the circadian rhythm and the flowering pathways were coordinately involved in regulation of the male sterility transition.

## Methods

### Plant materials

The varieties Nongken 58 and the mutant Nongken 58S (*Oryza sativa *L. ssp. *japonica*) were used for this study. All plants were grown under natural conditions at Wuhan University Campus. SD (10-h light/14-h dark) treatment was conducted when the seedlings had greater than five leaves. Differentiation from vegetative growth to reproductive growth was promoted after 10-12 days of SD treatment. When the inflorescences had developed to the secondary branch differentiation stage, the rice plants were divided into two groups for LD (15-h light/9-h dark) and SD treatment, respectively.

### RNA isolation and cDNA synthesis

For total RNA isolation, the second fully expanded leaf was harvested at two developmental stages: glume primordium differentiation and pistil/stamen primordium formation. For diurnal expression profile analysis, the leaves were harvested at 4 h intervals over 1 day, frozen immediately in liquid nitrogen, and stored at -80°C until use [39]. The samples are annotated in Additional file 1, Table S1.

A TRIzol Reagent Kit (Invitrogen, Carslbad, USA) was used for total RNA isolation following the manufacturer's instructions. Total RNA was treated with RNase-free DNase I (New England Biolabs, Hitchin, UK) to remove DNA contamination before cDNA synthesis. Two micrograms of total RNA and Oligo (dT) were used as template and primers for first strand cDNA synthesis by M-MLV reverse transcriptase (Promega, Madison, USA).

### Microarray analysis procedure and comparison strategy

Two micrograms of total RNA were used for double-stranded cDNA synthesis, and biotin-tagged cRNA was prepared using a MessageAmp™ II cRNA Amplification Kit according to the manufacturer's instructions. The resulting bio-tagged cRNA was fragmented into strands of 35-200 bases according to Affymetrix protocols. The fragmented cRNA was hybridised to an Affymetrix GeneChip Rice Genome Array containing 48,564 and 1,260 transcripts representing the *japonica *and *indica *cultivars, respectively. The microarray and data analysis were contracted to CapitalBio Corporation (Beijing, China). The hybridisation was performed at 45°C with rotation for 16 h (GeneChip Hybridization Oven 640, Affymetrix). The GeneChip arrays were washed and then stained (streptavidin-phycoerythrin) on an Affymetrix Fluidics Station 450. Scanning was conducted using a GeneChip Scanner 3000. All chip data represented biological triplicates.

The scanned images were examined by visual inspection and then processed to generate raw data using the default setting of GeneChip Operating Software (GCOS 1.4). dChip software was used to perform invariant-set normalization to normalize the arrays according to dChip users' manual. 5% of Perfect-Match (PM) probe signals were used as background removal. Annotation of the samples is shown in Additional file 1, Table S1. The samples were compared as follows: G-SSD2 vs. G-SLD2 and P-SSD2 vs. P-SLD2. The criteria for determining up- and down-regulated genes were fold changes (FCs) of ≥ 2 and ≤ 0.5, respectively. The complete microarray data have been deposited in NCBI's Gene Expression Omnibus and are accessible through GEO series accession number GSE29820.

### Cluster, gene ontology annotation and pathway analysis

All microarray data were analysed using Significant Analysis of Microarray (SAM) 3.02 software. Genes with FC ≥ 2 or FC ≤ 0.5 were chosen for the t-test, and genes with *P *values < 0.05 were chosen for further analysis. Cluster analysis was performed using Cluster 3.0 software. GO annotation was performed using the GeneOntology Enrichment Analysis Software Toolkit (http://omicslab.genetics.ac.cn/GOEAST/php/affymetrix.php) [40,41]. GOs with *P *values < 0.01 were selected. Pathway analysis was accomplished using the MAS 3.0 system (http://www.capitalbio.com/index.asp) (CapitalBio Corporation, Beijing, China) and by searching the Kyoto Encyclopedia of Genes and Genomes, BioCarta and GenMAPP databases. Pathways with *P *values < 0.01 were selected.

### Quantitative PCR analysis

For quantitative PCR, the cycling conditions were 95°C for 10 min, followed by 40 cycles of 95°C for 10 s and 60°C for 30 s, and then a melting curve from 60°C to 95°C in 0.3°C increments. Negative controls without the template DNA were used to ensure that primer dimers were not interfering with amplification. qPCR data was captured and analysed using StepOne software (ver. 2.0). The relative gene expression levels in each cDNA sample were obtained by normalisation to *ACTIN1 *using the formula 2^-(CT gene - CT actin1) ^[42]. The primers used in this study are listed in Additional file 2, Table S2. All qPCR data is the result of biological triplicates.

## Results

### Dynamic transcriptome profiling of photoperiod treatment during the male sterility transition

**Figure 1:**
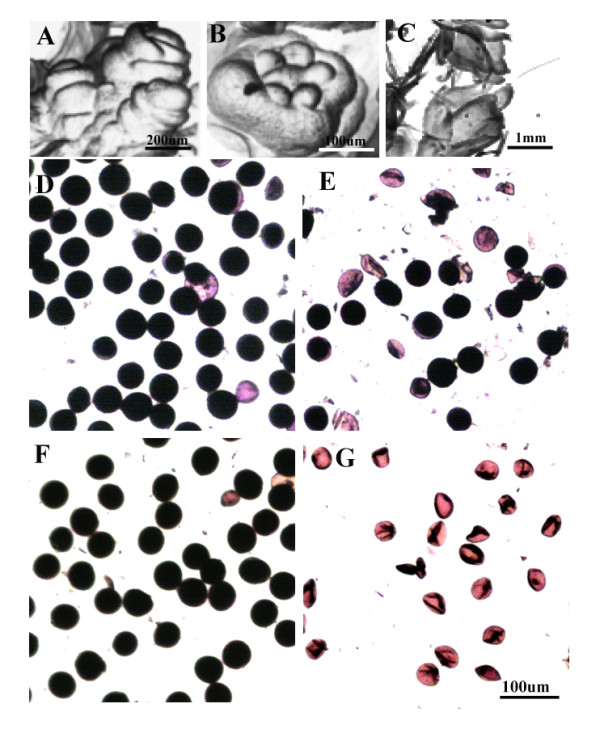
**Pollen fertility of Nongken 58S and Nongken 58 under LD and SD conditions **Scanning microscope images of rice inflorescence developmental stages and the pollen fertility of Nongken 58 and Nongken 58S under LD and SD conditions. (A) glume primordium differentiation stage, Bar = 200 μm; (B) pistil/stamen primordium forming stage, Bar = 100 μm; (C) pollen mother cell forming stage, Bar = 1 mm; Pollen fertility of Nongken 58 (D) and Nongken 58S (E) under SD conditions. Pollen fertility of Nongken 58 (F) and Nongken 58S (G) under LD conditions Bar = 100 μm; D, E, F have the same magnification as G. Pollen was stained with 1% I_2_/KI and observed under a light microscope.

As shown in Figure 1, pollen abortion was induced in Nongken 58S under LD conditions (15-h light/9-h dark) from the glume primordium differentiation to the pollen mother cell forming stages (Figure 1A-C) [2,43]. Under SD conditions (10-h light/14-h dark), the pollen partially regained fertility (Figure 1E, G). By contrast, pollen fertility in Nongken 58 was not affected (Figure 1D, F). These results indicated that the fertility of PGMS could not be completely restored using normal photoperiod conditions, implying that some defective signaling pathway may be constitutively affected. To investigate the transcriptome changes during the male sterility transition process in PGMS rice, we chose the leaves, light signal receiver, as experimental material at photoperiod-sensitive stage. Based on preliminary experiments, the diurnal expression profiles of those genes peaked at 02:00 h (data not shown). Thus, 02:00 h was selected as the expression profile time point for the microarray. Affymetrix GeneChips were used for global transcriptome profiling analysis. The correlation coefficients between different biological repeats were calculated, and the result indicates that the profilings were reproducible (Additional file 3, Table S3). A total of 20,444 and 19,964 transcripts were detected *P *values < 0.05 in the comparison of G-SSD2 vs. G-SLD2 and P-SSD2 vs. P-SLD2, respectively. In comparing the SD and LD conditions at the two developmental stages, 183 DEGs with two fold changes were detected *P *< 0.05.

**Figure 2:**
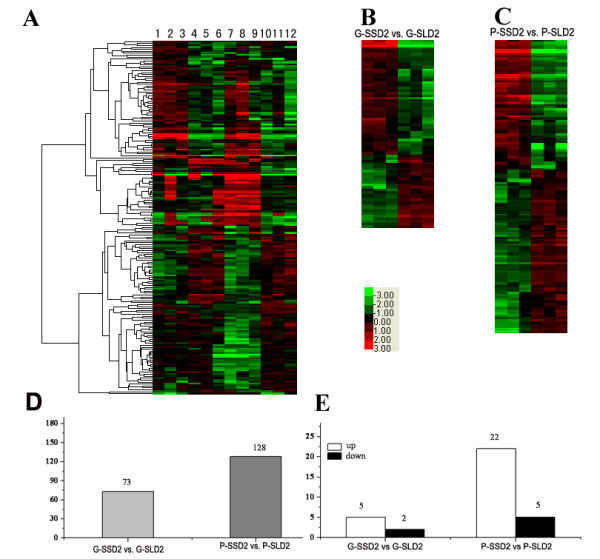
**Clustering analysis of DEGs **(A) Clustering of 183 DEGs with biological triplicates; (B) clustering of DEGs in G-SSD2 vs. G-SLD2; (C) clustering of the DEGs in P-SSD2 vs. P-SLD2; (D) diagram of the DEGs in both developmental stages; and (E) a diagram of up- and down- regulated genes. The scale bar represents the levels of up- (red) and down- (green) regulation.

The DEGs were categorised using Cluster 3.0 software (Figure 2A, B, C). The number of DEGs with FC ≥ 2 or ≤ 0.5 increased significantly as inflorescence development proceeded (Figure 2D, E; Additional file 4, Table S4; Additional file 5, Table S5). At the glume primordium differentiation and the pistil/stamen primordium forming stages, 73 and 128 DEGs with FC ≥ 2 or ≤ 0.5 (*P *< 0.05) were detected, respectively (Figure 2D). Five genes were identified as markedly up-regulated (≥ 5-fold) and two genes were noted as considerably down-regulated (≥ 5-fold) under SD conditions at the glume primordium stage (Figure 2E; Additional file 4, Table S4). Twenty-two genes were considerably up-regulated (≥ 5-fold), and only five genes were down-regulated (≥ 5-fold) at the pistil/stamen primordium forming stage (Figure 2E, Additional file 5, Table S5). These results indicated that more genes were suppressed than were activated under LD conditions. This suggests that transcriptome profiling in Nongken 58S was significantly suppressed, and the repression effects were markedly intensified as inflorescence development proceeded. This is consistent with previous studies in which pollen fertility was found to decrease as LD treatment increased [44].

### Defining DEGs for GO annotation and pathway analysis

To annotate the DEGs, the sequences were uploaded to the GOEAST website for GO annotation analysis [40,41,45]. The results indicated that some biological processes were significantly affected during the second photoperiod under LD conditions. Four GO biological processes related to the regulation of the transition from the vegetative to the reproductive phase (GO:0048510), the regulation of SD photoperiodism (GO:0048572), the photoperiodism (GO:0009648), and inflorescence development (GO:0010229) were investigated during the second photoperiod (Table 1). The results implied that these processes were related to the male sterility transition in PGMS rice.

**Table 1:** GO biological process of DEGs^1^

GO term	**Gene number**.	***P *value**^**2**^	*Q *value
**G-SSD2 vs. G-SLD2**			
GO:0006350 transcription	8	0.0000034	0.0000562
GO:0048510 regulation of timing of transition from vegetative to reproductive phase	1	0.000759	0.004176
GO:0048572 short-day photoperiodism	1	0.000759	0.004176
GO:0009648 photoperiodism	1	0.001518	0.005566
GO:0010229 inflorescence development	1	0.002276	0.007426
GO:0006950 response to stress	6	0.005823	0.012811
GO:0009628 response to abiotic stimulus	5	0.006268	0.012837
GO:0006352 transcription initiation	1	0.007944	0.014171
GO:0006265 DNA topological change	1	0.007944	0.014171
**P-SSD2 vs. P-SLD2**			
GO:0048510 regulation of transition from vegetative to reproductive phase	1	0.001156	0.021567
GO:0048572 short-day photoperiodism	1	0.001156	0.021567
GO:0009648 photoperiodism	1	0.002311	0.021567
GO:0010229 inflorescence development	1	0.003464	0.02425

^1^DEGs with FC ≥ 2 or ≤ 0.5 and *P *< 0.05 were chosen for GO annotation.

^2^GO terms with *P *< 0.01 are listed in the table.

To define the potential signal transduction pathways, the DEGs were uploaded to the MAS 3.0 system. Five pathways and seven pathways were identified from the transcriptome data for G-SSD2 vs. G-SLD2 and P-SSD2 vs. P-SLD2, respectively (Table 2). The circadian rhythm pathway was top ranked (*P *= 6.57E-06 and 1.38E-05) in both inflorescence developmental stages (Table 2). The results indicated that three genes, *OsPRR1 *(homologous to *PRR1*/*TOC1 *in *Arabidopsis*), *OsLHY *(homologous to *LHY *in *Arabidopsis*) and *MYB *transcription factor (LOC_Os06g51260), belong to the circadian rhythm pathway. This suggests that the circadian rhythm may be a principal signal transduction pathway in the male sterility transition in PGMS.

**Table 2:** Signal transduction pathway analysis of DEGs^1^

KEGG pathway	Gene number	***P *value**^**2**^	*Q *value
**G-SSD2 vs. G-SLD2**			
Circadian rhythm	2	0.00000657	0.0000131
Thiamine metabolism	1	0.000573	0.000573
Streptomycin biosynthesis	1	0.001065	0.00071
Inositol phosphate metabolism	1	0.006783	0.003235
Starch and sucrose metabolism	1	0.008086	0.003235
**P-SSD2 vs. P-SLD2**			
Circadian rhythm	2	0.0000138	0.0000276
Thiamine metabolism	1	0.000803	0000803
Cyanoamino acid metabolism	1	0.002521	0.001318
Ubiquinone biosynthesis	1	0.002636	0.001318
Nitrogen metabolism	1	0.004123	0.001412
Galactose metabolism	1	0.004237	0.001412
Alanine and aspartate metabolism	1	0.005494	0.00157

^1^DEGs with FC ≥ 2 or ≤ 0.5 and *P *< 0.05 were chosen for pathway analysis.

^2 ^Pathways with *P *< 0.01 are listed in the table.

### Circadian rhythm involvement in the male sterility transition

As mentioned previously, several clock genes [*OsPRR1 *(LOC_Os02g40510), *OsPRR37 *(LOC_Os07g49460), *OsGI *(LOC_Os01g08700), *OsLHY *(LOC_Os08g06110) and *MYB *transcription factor (LOC_Os06g51260)] were identified (Additional file 4, Table S4, Additional file 5, Table S5). Among these genes, *OsPRR37 *and *OsGI *were up-regulated 3.0-fold and 8.3-fold, respectively; *OsLHY *and the *MYB *transcription factor were down-regulated 2.4-fold and 2.6-fold, respectively. No difference in *OsPRR1 *expression was detected in the G-SSD2 vs. G-SLD2 data (Additional file 4, Table S4); in the P-SSD2 vs. P-SLD2 data, *OsPRR1 *was up-regulated 2.0-fold and the *MYB *transcription factor was down-regulated 4.4-fold. No differences in *OsPRR37, OsGI *and *OsLHY *were detected at this inflorescence developmental stage. The fold changes indicate that these clock genes were activated or repressed under LD conditions in Nongken 58S, implying that they may participate in the male sterility transition.

**Figure 3:**
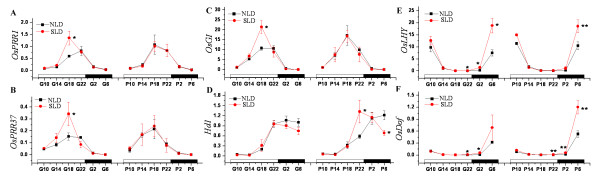
**Diurnal expression profiles of *OsPRR1, OsPRR37, OsGI, Hd1, OsLHY *and *OsDof ***The diagram shows the circadian rhythms of *OsPRR1, OsPRR37, OsGI, Hd1, OsLHY *and *OsDof *under LD conditions. The y-axis represents the expression ratio relative to the endogenous reference *Actin1 *gene using qPCR. The boxes on the x-axis represent day (blank) and night (solid). NLD and SLD represent Nongken 58 and Nongken 58S under LD conditions. G and P indicate the glume primordium differentiation and pistil/stamen primordium forming stages, respectively. Asterisks show statistically significant differences (* = *P *< 0.05; ^** ^= *P *< 0.01).

Because gene transcription is dynamic, the microarray data reflect expression differences at 02:00 h only. Therefore, the diurnal expression profiles of these genes were analysed in both Nongken 58S and Nongken 58 at the glume primordium differentiation and pistil/stamen primordium forming stages under LD and SD conditions. *OsPRR1, OsPRR37 *and *OsGI *exhibited the similar circadian rhythm patterns under LD conditions (Figure 3A-C). For three of these genes, the differences between Nongken 58S and Nongken 58 only occurred at 18:00 h at the glume primordium differentiation stage. The expression levels in Nongken 58S were significantly higher than in Nongken 58. No difference was found at the pistil/stamen primordium forming stage under LD and SD conditions (Figure 3A-C, Additional file 6, Figure S1A-C). These results indicate a circadian rhythm difference in the expression of the three genes between Nongken 58S and Nongken 58 [12].

*OsLHY*, a dawn-phased gene, is repressed during the day and activated at night. Its expression level differed significantly between Nongken 58S and Nongken 58 at 22:00 h, 02:00 h, 06:00 h at the glume primordium differentiation stage, and 06:00 h at the pistil/stamen primordium forming stage (Figure 3E). However, no difference was detected under SD conditions (Additional file 6, Figure S1E). The results also indicated circadian rhythm differences between Nongken 58S and Nongken 58. *OsDof *(LOC_Os01g15900), a Dof-type zinc finger (Additional file 4, Table S4), showed a similar diurnal pattern to *OsLHY*. The major differences between Nongken 58S and Nongken 58 occurred at 22:00 h and 02:00 h at the glume primordium differentiation stage, and at 22:00 h, 02:00 h, and 06:00 h at the pistil/stamen primordium forming stage (Figure 3F).

Taken together, these differences only occurred under LD conditions at the photoperiod-sensitive developmental stage. Based on the genetic background of Nongken 58S and Nongken 58, these genes were all related to the male sterility transition and the rice circadian rhythm was involved in this process.

### Flowering gene *Hd1 *is involved in the male sterility transition under LD conditions

*Ehd1, Hd3a, RFT1 *and *OsMADS1 *were all significantly suppressed under LD conditions in Nongken 58S (Additional file 4, Table S4; Additional file 5, Table S5). *Ehd1, Hd3a, RFT1 *are crucial genes for rice flowering [18-21,23], and *OsMADS1 *plays key roles in specifying floral organ and meristem identity in rice [46-48]. *Ehd2 *and *Hd1 *are also important components of the flowering pathway. To determine whether these genes were involved in the male sterility transition, the diurnal expression profiles of these genes were analysed. *Hd1 *was the only gene exhibiting different daily expression profiles between Nongken 58S and Nongken 58 under LD conditions (Figure 3D and Additional file 7, Figure S2). The main differences occurred at the pistil/stamen primordium forming stage. The transcription levels at 22:00 h and 06:00 h in Nongken 58S were significantly different from those in Nongken 58, indicating that the peak phase of *Hd1 *was at 06:00 h in Nongken 58 and it was shifted forward to 22:00 h in Nongken 58S. The phase is a mathematical term of plant circadian rhythms [12]. The phase-shift indicated that the circadian rhythm of *Hd1 *in Nongken 58S was notably different from that in Nongken 58, while the flowering time of Nongken 58 is similar to that of Nongken 58S under LD conditions. This suggests that *Hd1 *plays a role in the male sterility transition in PGMS.

### *OsPRR37 *and *Hd1 *were differentially expressed in the inflorescences of Nongken 58S and Nongken 58

**Figure 4:**
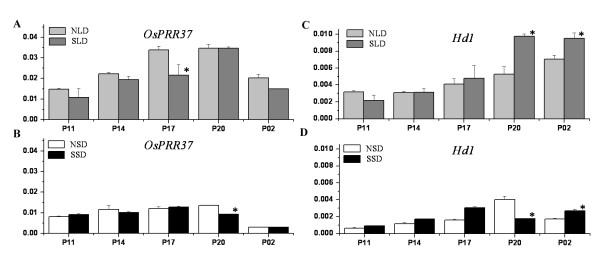
**Differential expressions of *OsPRR37 *and *Hd1 *in inflorescences **The bar chart shows the expression patterns of *OsPRR37 *and *Hd1 *under LD and SD conditions. The y-axis represents the expression ratio relative to the endogenous reference *Actin1 *gene using qPCR. NLD and SLD represent Nongken 58 and Nongken 58S under LD conditions. NSD and SSD represent Nongken 58 and Nongken 58S under SD conditions. P represents pistil/stamen primordium forming stages. Asterisks show statistically significant differences (* = *P *< 0.05).

Because of light signals received from the leaf, knowledge of the expression profiles of DEGs in the inflorescence is essential. Further qPCR of the expression patterns of these genes in the inflorescence showed that *OsPRR1, OsGI, OsLHY, OsDof *expression was similar in Nongken 58S and Nongken 58 under LD and SD conditions (Additional file 8, Figure S3). Only *OsPRR37 *and *Hd1 *were differentially expressed in the inflorescences of Nongken 58S and Nongken 58 at the pistil/stamen primordium forming stage (Figure 4). The expression level of *OsPRR37 *in Nongken 58S was significantly lower than that in Nongken 58 at 17:00 h under LD conditions (Figure 4A), and the level of *OsPRR37 *was significantly lower than that in Nongken 58 at 20:00 h under SD conditions (Figure 4B). The expression level of *Hd1 *in Nongken 58S was significantly greater than that in Nongken 58 at 20:00 h and 02:00 h under LD conditions (Figure 4C), but was significantly lower than that in Nongken at 20:00 h. However, the transcription level in Nongken 58S at 02:00 h was higher than that in Nongken 58. These results suggest that *OsPRR37 *and *Hd1 *are likely involved in the male sterility transition directly under LD conditions in the inflorescence. Moreover, the differences under SD conditions suggest a potential explanation for why pollen fertility cannot be completely recovered under SD in Nongken 58S.

## Discussion

Circadian rhythm and light signals have been reported to be involved in several physiological processes, including flowering, various stress responses and metabolism [26,30,31,38]. However, this is the first report suggesting a role for circadian rhythm and flowering signals in the regulation network of male sterility. In general, these genes promote the transition from vegetative growth to reproductive growth under SD conditions in SD plants and under LD conditions in LD plants [23]. Although the male sterility transition in PGMS rice is known to be regulated by LD conditions, the mechanism of regulation remains unknown. Global gene expression profiling under LD conditions revealed a suppressive trend and categorised into the circadian rhythm and flowering pathways. Both pathways regulate the process in a coordinated manner. Further studies revealed that the diurnal expression profiles of *OsPRR1, OsPRR37, OsGI, OsLHY, OsDof *and *Hd1 *under LD conditions were reprogrammed.

In this study, *OsPRR1, OsPRR37 *and *OsGI *exhibited similar expression patterns in the leaf under LD conditions, suggesting that these three genes may function together. *OsLHY *and *OsDof *also exhibit similar differentially expressed patterns, suggesting that they may function coordinately. In *Arabidopsis, TOC1*/*PRR1, LHY *and *GI*, key component factors in the central clock, comprise interlocked transcriptional feedback loops to regulate plant circadian rhythms [12]. In PGMS rice, *OsPRR1, OsLHY *and *OsGI *were not only involved in the circadian rhythm, but were also involved in the male sterility transition in coordination with *OsPRR37 *and *OsDof*. The LD signals maybe integrated and transmitted to the downstream genes through rice central clock in leaves. The day length signal may have been transmitted to the inflorescence via *Hd1 *and *OsPRR37*, ultimately leading to pollen abortion.

In the rice flowering pathway, *OsGI *is upstream of *Hd1 *and is positively correlated with *Hd1 *expression. *Hd1 *is activated by *OsGI *under LD or SD conditions. *Hd1 *activates *Hd3a*/*RFT1 *expression under SD conditions to promote flowering. However, *Hd1 *suppresses *Hd3a*/*RFT1 *expression under LD conditions to inhibit flowering [24]. In PGMS rice, *Hd1 *expression may also have a positive correlation with *OsGI *under LD conditions in Nongken 58S, as its expression was activated after *OsGI *expression (Figure 3 C, D). This implies that *Hd1 *may function downstream of *OsGI *in the male sterility transition in PGMS rice. In this case, *Hd3a*/*RFT1 *may not be the downstream regulator of *Hd1 *since there were no rhythm differences between Nongken 58S and Nongken 58. *Hd1 *was activated by *OsGI *under LD conditions and suppressed the unknown factors. This may influence downstream gene expression and finally affect male fertility.

In leaves, the diurnal expression patterns of *OsPRR1, OsPRR37, OsGI, OsLHY, OsDof *and *Hd1 *showed significant differences between Nongken 58S and Nongken 58. In the inflorescence, only *OsPRR37 *and *Hd1 *exhibited different expression patterns between Nongken 58S and Nongken 58 under both LD and SD. Therefore, it is speculated that the clock genes in leaves may function as sensors for day length. These genes may receive day length signals and integrate and transmit the signals into the inflorescence through a series of unknown pathways. Both *OsPRR37 *and *Hd1 *may be the effectors in the inflorescence, and they are likely involved in regulation of male sterility directly.

Previous studies have indicated that phytochromes and cryptochromes are involved in the male sterility transition. However, there were no diurnal expression differences in *OsPhyA, OsPhyB, OsCry1a, OsCry1b, OsCry2 *and *OsCry3 *under LD conditions between Nongken 58S and Nongken 58 (data not shown). Using an ELISA assay, Wang, et al. found that phyA content in Nongken 58S leaves was higher than in Nongken 58 under the identical day length treatment [49]. The differences in phytochromes and cryptochromes may be at the protein level and needs further investigation.

## Conclusion

In conclusion, the transcriptome of Nongken 58S was significantly suppressed and the repression effects were markedly intensified when inflorescence development proceeded. Rice circadian rhythm genes *OsPRR1, OsPRR37, OsGI, OsLHY *and *OsDof *and the flowering gene *Hd1 *were coordinately involved in signal transduction in leaves. Furthermore, *Hd1 *and *OsPRR37 *may be signal sensors in inflorescences to directly affect the male sterility transition.

## Authors' contribution

WW, ZL, ZG, GS, QC and DY conceived and designed the experiments; WW and ZL analysed the microarray data; WW, ZL, ZG, GS, DJ conducted portions of the experiments; WW, ZL, ZG, GS, YZ and DY prepared the manuscript. All authors have read and approved the final manuscript.

## Supplementary Material

Additional file 1 **Table S1 Annotation of sample names**. G stands for glume primordium differentiation stage; P stands for pistil/stamen primordium forming stage; S stands for Nongken 58S; SD is the abbreviation for short day condition and LD is the abbreviation for long day condition; 2 stands for 02:00 h. Click here for file

Additional file 2 **Table S2 Primers used for qPCR**. All of these primers were designed by Primer Express 3.0. Click here for file

Additional file 3 **Table S3 The Correlation coefficients between different biological repeats**. The correlation coefficients are calculated by Prism 5.0 software. 1,2,3 represent three different biological repeats. Click here for file

Additional file 4 **Table S4 Differentially expressed genes in G-SSD2 vs. G-SLD2 data**. All the DEGs with FC ≥ 2 or FC ≤ 0.5 are listed in this table. Click here for file

Additional file 5 **Table S5 Differentially expressed genes in P-SSD2 vs. P-SLD2 data**. All the DEGs with FC ≥ 2 or FC ≤ 0.5 are listed in this table. Click here for file

Additional file 6 **Figure S1 Diurnal expression profiles of *OsPRR1, OsPRR37, OsGI, Hd1, OsLHY *and *OsDof *genes in leaf**. The diagram shows the diurnal expression profiles of *OsPRR1, OsPRR37, OsGI, Hd1, OsLHY *and *OsDof *genes under SD conditions. The y-axis represents the expression ratio relative to the endogenous reference *Actin1 *gene using qPCR. The boxes on the x-axis represent day (blank) and night (solid). NSD and SSD represent Nongken 58 and Nongken 58S under SD conditions. G and P indicate the glume primordium differentiation and pistil/stamen primordium forming stages, respectively. Click here for file

Additional file 7 **Figure S2 Diurnal expression profiles of *Ehd2, Ehd1, Hd3a, RFT1 *and *OsMADS1 *under LD conditions in leaf**. The diagram shows the circadian rhythm of the expression of *Ehd2, Ehd1, Hd3a, RFT1 *and *OsMADS1 *under LD conditions. The y-axis represents the expression ratio relative to the endogenous reference *Actin1 *gene using qPCR. The boxes on the x-axis represent day (blank) and night (solid). NLD and SLD represent Nongken 58 and Nongken 58S under LD conditions. G and P indicate the glume primordium differentiation and pistil/stamen primordium forming stages, respectively. Click here for file

Additional file 8 **Figure S3 Expression patterns of *OsPRR1, OsGI, OsLHY *and *OsDof *under LD and SD conditions in inflorescence**. The bar chart shows the expression patterns of *OsPRR1, OsGI, OsLHY *and *OsDof *under LD and SD conditions in inflorescence. The y-axis represents the expression ratio relative to the endogenous reference *Actin1 *gene using qPCR. NLD and SLD represent Nongken 58 and Nongken 58S under LD conditions. NSD and SSD represent Nongken 58 and Nongken 58S under SD conditions. P indicates the pistil/stamen primordium forming stages. Asterisks show statistically significant differences (* = *P *< 0.05). Click here for file

## References

[B1] Shi MS The discovery and study of the photosensitive recessive male-sterile rice Scientia Agricultura Sinica 1985 2 44 48

[B2] Yuan SC Zhang ZG Xu CZ Studies on the critical stage of fertility change induced by light and its phase development in HPGMR Acta Agronomica Sinica 1988 14 7 13

[B3] Li XH Wang FL Lu Q Xu CG Fine Mapping of PSGMS Gene pms3 in Rice Acta Agronomica Sinica 2002 28 310 314

[B4] Mei MH Chen L Zhang Z Li Z Xu C Zhang Q pms3 is the locus causing the original photoperiod-sensitive male sterility mutation of 'Nongken 58S' Science China Life Sciences 1999 42 316 322 10.1007/BF03183609 20229347

[B5] Wang FP Mei MH Xu CG Zhang QF pms1 is not the Locus Relevant to Fertility Difference between the Photoperiod-sensitive Male Sterile Rice Nongken 58S and Normal Rice "Nongken 58" Acta Botanica Sinica 1997 39 922 925

[B6] Zhang Q Shen BZ Dai XK Mei MH Saghai Maroof MA Li ZB Using bulked extremes and recessive class to map genes for photoperiod-sensitive genic male sterility in rice Proceedings of National Academic of Science 1994 91 8675 8679 10.1073/pnas.91.18.8675 PMC44669 7915844

[B7] Zhang XG Zhu YG A Genetic Study on Sterility of Hubei Photoperiod Sensitive Genic Male-Sterile Rice Journal of Huazhong Agricultural University 1990 9 481 483

[B8] Liu N Shan Y Wang FP Xu CG Peng KM Li XH Zhang QF Identification of an 85-kb DNA fragment containing pms1, a locus for photoperiod-sensitive genic male sterility in rice Molecular Genetics and Genomics 2001 266 271 275 10.1007/s004380100553 11683269

[B9] Lu Q Li XH Guo D Xu CG Zhang Q Localization of pms3, a gene for photoperiod-sensitive genic male sterility, to a 28.4-kb DNA fragment Molecular Genetics and Genomics 2005 273 507 511 10.1007/s00438-005-1155-4 15912317

[B10] Dunlap JC Loros JJ DeCoursey P Chronobiology: Biological Timekeeping 2004

[B11] McClung CR Plant circadian rhythms Plant Cell 2006 18 792 803 10.1105/tpc.106.040980 16595397 PMC1425852

[B12] Harmer SL The circadian system in higher plants Annual Review of Plant Biology 2009 60 357 377 10.1146/annurev.arplant.043008.092054 19575587

[B13] Somers DE Webb AA Pearson M Kay SA The short-period mutant, toc1-1, alters circadian clock regulation of multiple outputs throughout development in Arabidopsis thaliana Development 1998 125 485 494 9425143 10.1242/dev.125.3.485

[B14] Schaffer R Ramsay N Samach A Corden S Putterill J Carre IA Coupland G The late elongated hypocotyl mutation of Arabidopsis disrupts circadian rhythms and the photoperiodic control of flowering Cell 1998 93 1219 1229 10.1016/S0092-8674(00)81465-8 9657154

[B15] Wang ZY Tobin EM Constitutive expression of the CIRCADIAN CLOCK ASSOCIATED 1 (CCA1) gene disrupts circadian rhythms and suppresses its own expression Cell 1998 93 1207 1217 10.1016/S0092-8674(00)81464-6 9657153

[B16] Locke JC Southern MM Kozma-Bognar L Hibberd V Brown PE Turner MS Millar AJ Extension of a genetic network model by iterative experimentation and mathematical analysis Molecular Systems Biology 2005 1 2005 0013 16729048 10.1038/msb4100018 PMC1681447

[B17] Locke JC Kozma-Bognar L Gould PD Feher B Kevei E Nagy F Turner MS Hall A Millar AJ Experimental validation of a predicted feedback loop in the multi-oscillator clock of Arabidopsis thaliana Molecular Systems Biology 2006 2 59 17102804 10.1038/msb4100102 PMC1682024

[B18] Komiya R Yokoi S Shimamoto K A gene network for long-day flowering activates RFT1 encoding a mobile flowering signal in rice Development 2009 136 3443 3450 10.1242/dev.040170 19762423

[B19] Komiya R Ikegami A Tamaki S Yokoi S Shimamoto K Hd3a and RFT1 are essential for flowering in rice Development 2008 135 767 774 10.1242/dev.008631 18223202

[B20] Tamaki S Matsuo S Wong HL Yokoi S Shimamoto K Hd3a protein is a mobile flowering signal in rice Science 2007 316 1033 1036 10.1126/science.1141753 17446351

[B21] Doi K Izawa T Fuse T Yamanouchi U Kubo T Shimatani Z Yano M Yoshimura A Ehd1, a B-type response regulator in rice, confers short-day promotion of flowering and controls FT-like gene expression independently of Hd1 Genes & Development 2004 18 926 936 10.1101/gad.1189604 15078816 PMC395851

[B22] Kojima S Takahashi Y Kobayashi Y Monna L Sasaki T Araki T Yano M Hd3a, a rice ortholog of the Arabidopsis FT gene, promotes transition to flowering downstream of Hd1 under short-day conditions Plant & Cell Physiology 2002 43 1096 1105 10.1093/pcp/pcf156 12407188

[B23] Izawa T Daylength measurements by rice plants in photoperiodic short-day flowering International Review of Cytology 2007 256 191 222 17241908 10.1016/S0074-7696(07)56006-7

[B24] Hayama R Yokoi S Tamaki S Yano M Shimamoto K Adaptation of photoperiodic control pathways produces short-day flowering in rice Nature 2003 422 719 722 10.1038/nature01549 12700762

[B25] Yano M Katayose Y Ashikari M Yamanouchi U Monna L Fuse T Baba T Yamamoto K Umehara Y Nagamura Y Sasaki T Hd1, a major photoperiod sensitivity quantitative trait locus in rice, is closely related to the Arabidopsis flowering time gene CONSTANS Plant Cell 2000 12 2473 2484 11148291 10.1105/tpc.12.12.2473 PMC102231

[B26] Turck F Fornara F Coupland G Regulation and identity of florigen: FLOWERING LOCUS T moves center stage Annual Review of Plant Biology 2008 59 573 594 10.1146/annurev.arplant.59.032607.092755 18444908

[B27] Kobayashi Y Weigel D Move on up, it's time for change-mobile signals controlling photoperiod-dependent flowering Genes & Development 2007 21 2371 2384 10.1101/gad.1589007 17908925

[B28] Dodd AN Salathia N Hall A Kevei E Toth R Nagy F Hibberd JM Millar AJ Webb AA Plant circadian clocks increase photosynthesis, growth, survival, and competitive advantage Science 2005 309 630 633 10.1126/science.1115581 16040710

[B29] Yakir E Hilman D Harir Y Green RM Regulation of output from the plant circadian clock FEBS Journal 2007 274 335 345 10.1111/j.1742-4658.2006.05616.x 17229141

[B30] McClung CR Gutierrez RA Network news: prime time for systems biology of the plant circadian clock Current Opinion in Genetics & Development 2010 20 588 598 10.1016/j.gde.2010.08.010 20889330 PMC3098449

[B31] de Montaigu A Toth R Coupland G Plant development goes like clockwork Trends in Genetics 2010 26 296 306 10.1016/j.tig.2010.04.003 20483501

[B32] Doherty CJ Kay SA Circadian control of global gene expression patterns Annual Review of Genetics 2010 44 419 444 10.1146/annurev-genet-102209-163432 20809800 PMC4251774

[B33] Covington MF Maloof JN Straume M Kay SA Harmer SL Global transcriptome analysis reveals circadian regulation of key pathways in plant growth and development Genome Biology 2008 9 R130 10.1186/gb-2008-9-8-r130 18710561 PMC2575520

[B34] Michael TP Breton G Hazen SP Priest H Mockler TC Kay SA Chory J A morning-specific phytohormone gene expression program underlying rhythmic plant growth PLoS Biology 2008 6 e225 10.1371/journal.pbio.0060225 18798691 PMC2535664

[B35] Mizuno T Yamashino T Comparative transcriptome of diurnally oscillating genes and hormone-responsive genes in Arabidopsis thaliana: insight into circadian clock-controlled daily responses to common ambient stresses in plants Plant & Cell Physiology 2008 49 481 487 10.1093/pcp/pcn008 18202002

[B36] Nozue K Covington MF Duek PD Lorrain S Fankhauser C Harmer SL Maloof JN Rhythmic growth explained by coincidence between internal and external cues Nature 2007 448 358 361 10.1038/nature05946 17589502

[B37] Niwa Y Yamashino T Mizuno T The circadian clock regulates the photoperiodic response of hypocotyl elongation through a coincidence mechanism in Arabidopsis thaliana Plant & Cell Physiology 2009 50 838 854 10.1093/pcp/pcp028 19233867

[B38] Roden LC Ingle RA Lights, rhythms, infection: the role of light and the circadian clock in determining the outcome of plant-pathogen interactions Plant Cell 2009 21 2546 2552 10.1105/tpc.109.069922 19789275 PMC2768925

[B39] Yang DC Zhu YG Primary study on total leaf RNA in PGMR at different photoperiod treatments and developmental stages Acta Genetica Sinica 1990 17 308 312

[B40] Hulsegge I Kommadath A Smits MA Globaltest and GOEAST: two different approaches for Gene Ontology analysis BMC Proceeding 2009 3 Suppl 4 S10 10.1186/1753-6561-3-s4-s10 PMC2712740 19615110

[B41] Zheng Q Wang XJ GOEAST: a web-based software toolkit for Gene Ontology enrichment analysis Nucleic Acids Research 2008 36 W358 363 10.1093/nar/gkn276 18487275 PMC2447756

[B42] Xue W Xing Y Weng X Zhao Y Tang W Wang L Zhou H Yu S Xu C Li X Zhang Q Natural variation in Ghd7 is an important regulator of heading date and yield potential in rice Nature Genetics 2008 40 761 767 10.1038/ng.143 18454147

[B43] Shi Y Zhao S Yao J Premature tapetum degeneration: a major cause of abortive pollen development in photoperiod sensitive genic male sterility in rice Journal of Integrative Plant Biology 2009 51 774 781 10.1111/j.1744-7909.2009.00849.x 19686374

[B44] Xue GX Zhao JZ A Preliminary Study on the Critical-Daylength Evoking the Photoperiodic Sensitive Male Sterility of Rice and Their Responses to Other Environmental Factors Acta Agronomica Sinica 1990 16 112 122

[B45] Hedegaard J Arce C Bicciato S Bonnet A Buitenhuis B Collado-Romero M Conley LN Sancristobal M Ferrari F Garrido JJ Methods for interpreting lists of affected genes obtained in a DNA microarray experiment BMC Proceeding 2009 3 Suppl 4 S5 10.1186/1753-6561-3-s4-s5 PMC2712748 19615118

[B46] Chen ZX Wu JG Ding WN Chen HM Wu P Shi CH Morphogenesis and molecular basis on naked seed rice, a novel homeotic mutation of OsMADS1 regulating transcript level of AP3 homologue in rice Planta 2006 223 882 890 10.1007/s00425-005-0141-8 16254725

[B47] Agrawal GK Abe K Yamazaki M Miyao A Hirochika H Conservation of the E-function for floral organ identity in rice revealed by the analysis of tissue culture-induced loss-of-function mutants of the OsMADS1 gene Plant Mol Biol 2005 59 125 135 10.1007/s11103-005-2161-y 16217607

[B48] Prasad K Parameswaran S Vijayraghavan U OsMADS1, a rice MADS-box factor, controls differentiation of specific cell types in the lemma and palea and is an early-acting regulator of inner floral organs Plant J 2005 43 915 928 10.1111/j.1365-313X.2005.02504.x 16146529

[B49] Wang W Tong Z Kuang TY Tang PS Immunoassay of Phytochrome A Content in Photoperiod-sensitive Genic Male-sterile Rice Developmental and Reproductive Biology 1996 5 51 59

