# Supplementary material for: Comparative Transcriptomes Profiling of Photoperiod-sensitive Male Sterile Rice Nongken 58S During the Male Sterility Transition between Short-day and Long-day
Source: BMC Genomics. 2011 Sep 25;12:462. doi: 10.1186/1471-2164-12-462 (PMC3197534; doi:10.1186/1471-2164-12-462)
Supplement: Additional file 1 — Table S1 Annotation of sample names. G stands for glume primordium differentiation stage; P stands for pistil/stamen primordium forming stage; S stands for Nongken 58S; SD is the abbreviation for short day condition and LD is the abbreviation for long day condition; 2 stands for 02:00 h. [file 1471-2164-12-462-S1.DOC]

**Additional file 1 Table S1. The annotations of sample names**.

| Sample names | Annotations |
| --- | --- |
| G-SSD2 | Nongken58S leaf at Glume primordium differentiation stage under SD condition at 02:00 h |
| G-SLD2 | Nongken58S leaf at Glume primordium differentiation stage under LD condition at 02:00 h |
| P-SSD2 | Nongken58S leaf at Pistil/stamen primordium forming stage under SD condition at 02:00 h |
| P-SLD2 | Nongken58S leaf at Pistil/stamen primordium forming stage under LD condition at 02:00 h |

G stands for glume primordium differentiation stage; P stands for pistil/stamen primordium forming stage; S stands for Nongken 58S; SD is the abbreviation for short day condition and LD is the abbreviation for long day condition; 2 stands for 02:00 h.
