# Supplementary material for: Comparative Transcriptomes Profiling of Photoperiod-sensitive Male Sterile Rice Nongken 58S During the Male Sterility Transition between Short-day and Long-day
Source: BMC Genomics. 2011 Sep 25;12:462. doi: 10.1186/1471-2164-12-462 (PMC3197534; doi:10.1186/1471-2164-12-462)
Supplement: Additional file 2 — Table S2 Primers used for qPCR. All of these primers were designed by Primer Express 3.0. [file 1471-2164-12-462-S2.DOC]

**Additional file 2 Table S2. Primers used for qPCR**

| Gene | Sequence |
| --- | --- |
| *OsPRR1* | F: AGGGATGATGGACCACGGTAT  R: GGTGTATGCACTTGAGCGTTGT |
| *OsPRR37* | F: GAGGAACAACGGAATGTTTTGAG  R: TGGAAGCCACAGGTGTATGGTA |
| *OsGI* | F: TGGAAGTAACCGCCAGAGCAGTC  R: GTTGATAGACGGCACTTCAGCAG |
| *OsLHY* | F: CAGATAAGGCCGACACCAAAC  R: GGTGTGTTGGAACCACATG |
| LOC_Os06g51260 | F: GCGGCAGATGCTAGAATTCC  R: GGATGGCTAATGGGAGCAAA |
| *Ehd2* | F: CGACAATAGCTCGATCGCC  R: AAGCCCGAAGCTGACACTGT |
| *Hd1* | F: TCAGCAACAGCATATCTTTCTCATCA  R: TCTGGAATTTGGCATATCTATCACC |
| *Ehd1* | F: GCGCTTTTGATTTCCTGC  R: CGGAATATGTGCTGCCAA |
| *Hd3a* | F: GCTCACTATCATCATCCAGCATG  R: CCTTGCTCAGCTATTTAATTGCATAA |
| *RFT1* | F: CGTCCATGGTGACCCAACA  R: CCGGGTCTACCATCACGAGT |
| *OsMADS1* | F: CCCAGATCAGGGTGACCATT  R: GTCCATGTAGGCCTGGTGATG |
| *PHYA* | F: GCATTGGGGAAAACCTTCATCT  R: ACTCCTTTGCCCTGGTGCTT |
| *PHYB* | F: ATGGGTGGCGAGGTCCAATA  R: CCCCCTACTTGCTGCTTGCT |
| *CRY1a* | F: ACCGCATCGACAACCCTCA  R: AGCCATCGCCGGACATACT |
| *CRY1b* | F: TTTGAGCCTCGAACTGAGCG  R: GAGGTTGCTGGAAATTATTGTGC |
| *CRY2* | F: CTGGGAGCCTGCCTGATG  R: TTTGACCTTGAACCTCTGGATTATC |
| *CRY3* | F: CGTGGCCGTCAGATTGTCT  R: CCCATTCGCCAATCAATACC |
| *OsDof* | F: TTTCTGCACCACCATTTCCAT  R: GCCGGCCAGCTGGATAA |
| *Actin1* | F: GCCTTGGCAATCCACATC  R: AGCATGAAGATCAAGGTGGTC |

All of these primers were designed by Primer Express 3.0.
