# Supplementary material for: Comparative Transcriptomes Profiling of Photoperiod-sensitive Male Sterile Rice Nongken 58S During the Male Sterility Transition between Short-day and Long-day
Source: BMC Genomics. 2011 Sep 25;12:462. doi: 10.1186/1471-2164-12-462 (PMC3197534; doi:10.1186/1471-2164-12-462)
Supplement: Additional file 3 — Table S3 The Correlation coefficients between different biological repeats. The correlation coefficients are calculated by Prism 5.0 software. 1,2,3 represent three different biological repeats. [file 1471-2164-12-462-S3.DOC]

**Additional file 3 Table S3** **Correlation coefficients between different biological repeats**

| Microarray samples | Biological Correlation Coefficients | | |
| --- | --- | --- | --- |
|  | 1,2 | 2,3 | 1,3 |
| G-SSD2 | 0.9767591 | 0.9592376 | 0.9590073 |
| G-SLD2 | 0.9662216 | 0.9298014 | 0.953505 |
| P-SSD2 | 0.9515221 | 0.9296076 | 0.8924966 |
| P-SLD2 | 0.9648499 | 0.9175259 | 0.9374353 |

The correlation coefficients are calculated by Prism 5.0 software. 1,2,3 represent three different biological repeats.
