# Supplementary figures and images for: Comparative Transcriptomes Profiling of Photoperiod-sensitive Male Sterile Rice Nongken 58S During the Male Sterility Transition between Short-day and Long-day
Source: BMC Genomics. 2011 Sep 25;12:462. doi: 10.1186/1471-2164-12-462 (PMC3197534; doi:10.1186/1471-2164-12-462)

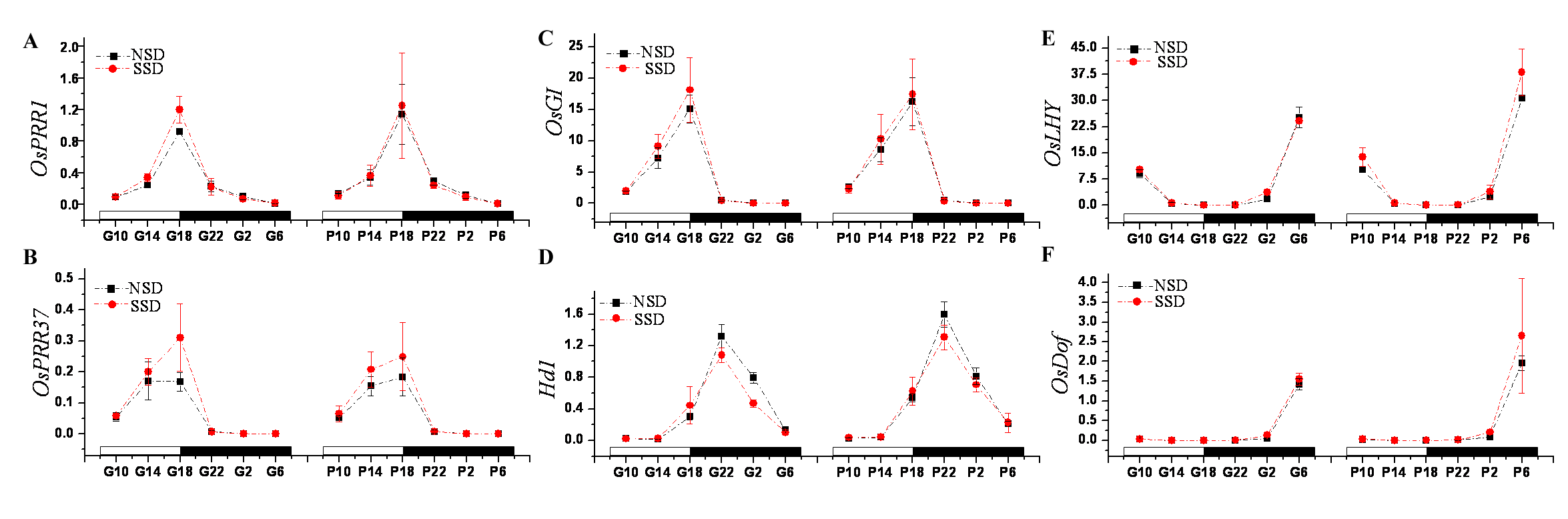

Supplement: Additional file 6 — Figure S1 Diurnal expression profiles of OsPRR1, OsPRR37, OsGI, Hd1, OsLHY and OsDof genes in leaf. The diagram shows the diurnal expression profiles of OsPRR1, OsPRR37, OsGI, Hd1, OsLHY and OsDof genes under SD conditions. The y-axis represents the expression ratio relative to the endogenous reference Actin1 gene using qPCR. The boxes on the x-axis represent day (blank) and night (solid). NSD and SSD represent Nongken 58 and Nongken 58S under SD conditions. G and P indicate the glume primordium differentiation and pistil/stamen primordium forming stages, respectively. [file 1471-2164-12-462-S6.TIFF]

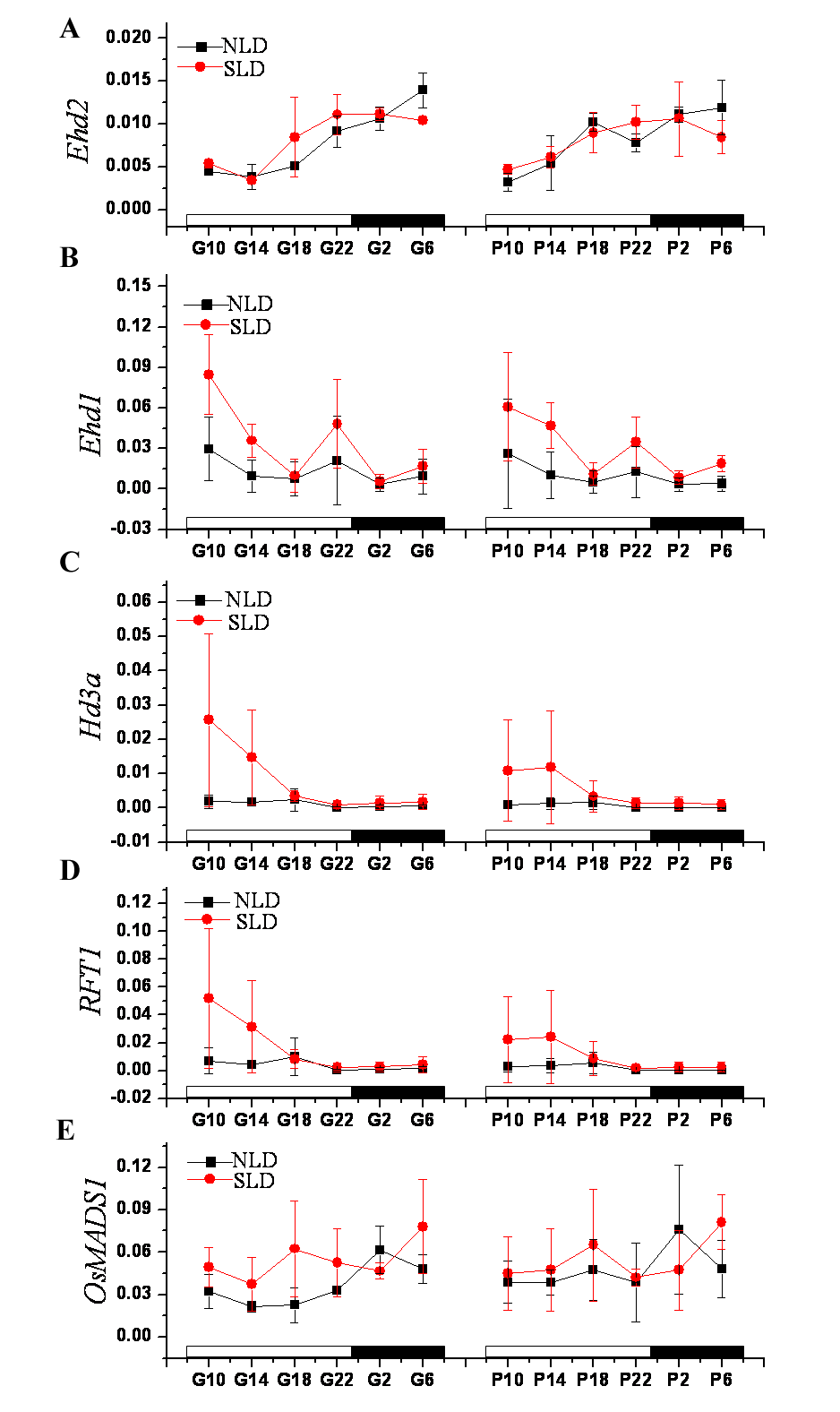

Supplement: Additional file 7 — Figure S2 Diurnal expression profiles of Ehd2, Ehd1, Hd3a, RFT1 and OsMADS1 under LD conditions in leaf. The diagram shows the circadian rhythm of the expression of Ehd2, Ehd1, Hd3a, RFT1 and OsMADS1 under LD conditions. The y-axis represents the expression ratio relative to the endogenous reference Actin1 gene using qPCR. The boxes on the x-axis represent day (blank) and night (solid). NLD and SLD represent Nongken 58 and Nongken 58S under LD conditions. G and P indicate the glume primordium differentiation and pistil/stamen primordium forming stages, respectively. [file 1471-2164-12-462-S7.TIFF]

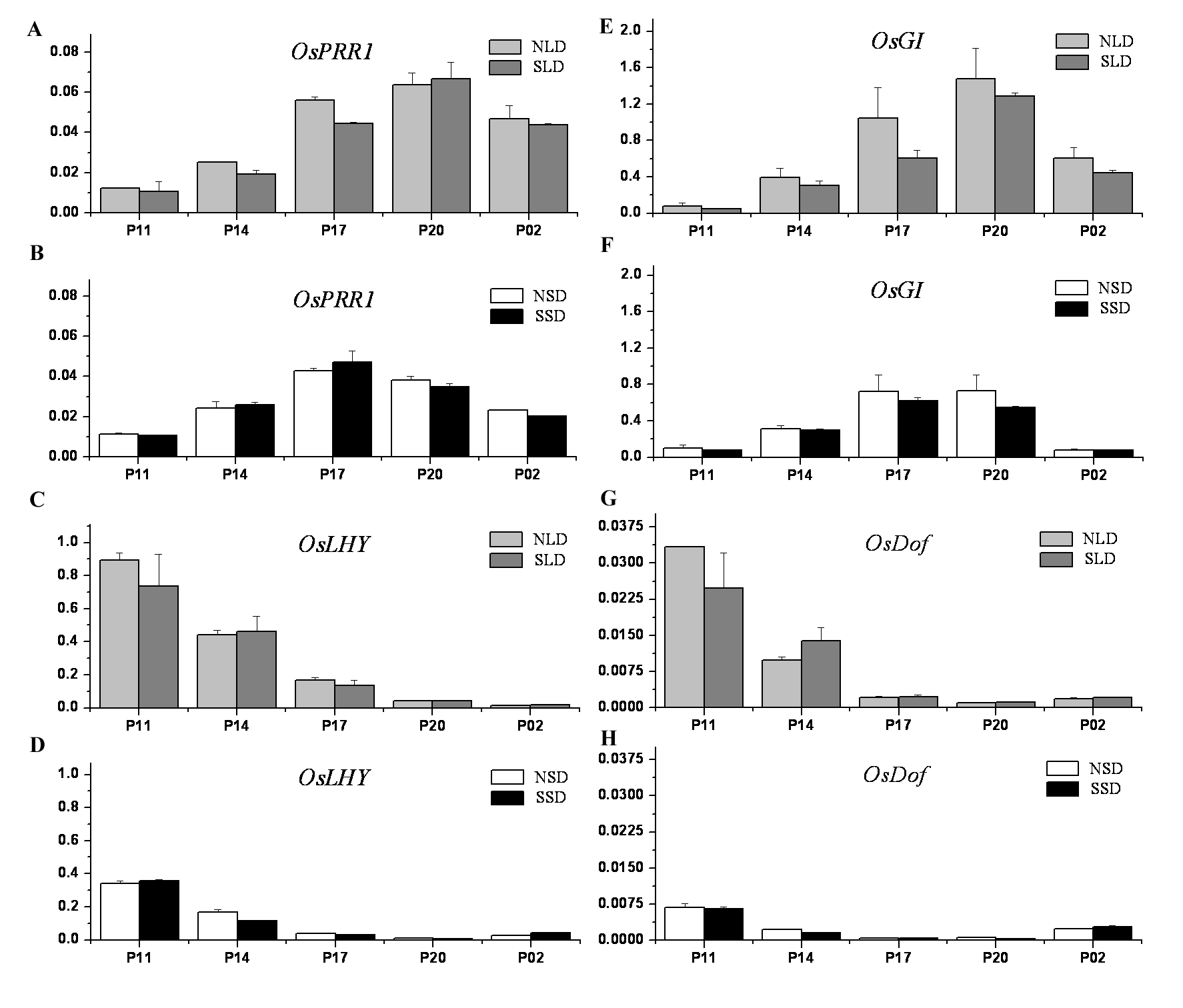

Supplement: Additional file 8 — Figure S3 Expression patterns of OsPRR1, OsGI, OsLHY and OsDof under LD and SD conditions in inflorescence. The bar chart shows the expression patterns of OsPRR1, OsGI, OsLHY and OsDof under LD and SD conditions in inflorescence. The y-axis represents the expression ratio relative to the endogenous reference Actin1 gene using qPCR. NLD and SLD represent Nongken 58 and Nongken 58S under LD conditions. NSD and SSD represent Nongken 58 and Nongken 58S under SD conditions. P indicates the pistil/stamen primordium forming stages. Asterisks show statistically significant differences (* = P < 0.05). [file 1471-2164-12-462-S8.TIFF]
